# The Impact of Information Presentation on Consumer Perceptions of Cricket-Containing Chocolate Chip Cookies

**DOI:** 10.3390/foods13030479

**Published:** 2024-02-02

**Authors:** Yupeng Gao, Pitchayapat Chonpracha, Bin Li, Ryan Ardoin, Witoon Prinyawiwatkul

**Affiliations:** 1 School of Nutrition and Food Sciences, Louisiana State University, Agricultural Center, Baton Rouge, LA 70803, USA; ygao19@lsu.edu (Y.G.); pitchayapat_chonpracha@hotmail.com (P.C.); 2 Department of Experimental Statistics, Louisiana State University, Agricultural Center, Baton Rouge, LA 70803, USA; bli@lsu.edu; 3 Food Processing and Sensory Quality Research Unit, Southern Regional Research Center, USDA-ARS, New Orleans, LA 70124, USA; ryan.ardoin@usda.gov

**Keywords:** entomophagy, cricket, consumer perception, informed conditions, willingness to consume, acceptance, purchase intent

## Abstract

As a source of protein and other nutrients for a growing population, edible insect production offers environmental and sustainability advantages over traditional meat production. Although around 2 billion people consume insects worldwide, Western consumers are still reluctant to practice entomophagy, hindered largely by neophobia and negative emotions. In addition to sensory quality and safety, an informational component may be crucial to consumers’ decision making involving insect consumption. In this study, three different information types, namely text, image, and a tangible product, were used to convey information about chocolate chip cookies (CCCs) containing cricket flour. The nature of the information was related to the ingredient usage level (5%), the type of insect (cricket), nutritional values, sustainability benefits, packaging, celebrity endorsement, and/or visual appearance of an actual product. Consumers’ willingness to consume (WTC), acceptance, and purchase intent (PI) were measured in response to each informed condition. Once informed of the insect ingredient, all scores significantly (α = 0.05) dropped. The lowest WTC (1.97 ± 1.06, Text), acceptance (3.55 ± 2.23, Image), and PI (1.85 ± 1.05, Text) scores were found after identifying cricket as the insect ingredient. Compared to other informed conditions, the presentation of a real chocolate chip cookie containing insects achieved the highest scores on all affective scores (WTC: 3.4 ± 1.04, acceptance: 6.17 ± 1.89, PI: 3.07 ± 1.09). The greatest improvement in scores was observed after information about nutrition and sustainability benefits (based on ANOVA), which was more impactful for males than females (based on a *t*-test). Celebrity endorsement did not have a significant effect. The presentation of the actual CCC containing cricket flour (for visual observation only) significantly increased WTC, acceptance, and PI compared to presenting text and images alone. Acceptance, WTC, and certain information cues were significant predictors of PI for CCCs containing cricket flour.

## 1. Introduction

Despite the potential benefits of sustainable nutrition, the reluctance of Western consumers to adopt entomophagy (consumption of insects) is well documented [1,2,3]. Edible insects encompass multiple species with various nutritional profiles and have been touted for complete protein, desirable fatty acid profiles, micronutrients, and bioactive compounds [4]. Compared to the traditional meat industry, food products that are made of edible insects may provide a more sustainable and environmentally friendly protein source and contribute to a circular economy [4,5]. However, food neophobia, perceived disgust, concerns about safety risks, and poor sensory quality are among the main reasons for widespread aversion among Western consumers [6,7]. Therefore, consumer science currently plays a crucial role in understanding and addressing barriers to entomophagy, if insects are to become a viable alternative and sustainable protein source globally.

One strategy to overcome psychological barriers to entomophagy has been the provision of product-related information or claims to potential consumers. Verbal descriptions of insect-containing foods employed in research have included positive messages about safety, sustainability, and/or nutritional benefits [8,9]. These rational appeals have demonstrated a positive effect on consumer attitudes and self-reported behavioral intent in general. However, the benefits of entomophagy on nutritional and environmental aspects are not always sufficient enough to reduce consumers’ negative emotional reactions, especially to “disgust” [7]. The health and sustainability claim also failed to increase the willingness to try when food neophobia exists [10].

Other studies have examined the effects of the images of products and packaging (e.g., photographs of the food item and/or graphics on labels) on the perceptions of insect-containing foods [11,12]. Insect-based food labeled from a positive image country contributes to higher quality expectations for consumers. This expectation helps to increase consumers’ intention to try insect-based food [13]. In general, consumers have responded more favorably to products when the insects are not visually detectable (or “invisible”) in the food, when insects are not depicted on the packaging, and when the product name does not directly identify the insect ingredient [2]. Additionally, a novel ingredient can be more acceptable when presented in a more familiar food [14]. Although consumers respond to text, images, and tangible products differently, little is known about how different combinations of these conditions affect perceptions of insect-based foods. In the present study, the effects of different verbal information cues (Text), pictures related to insect-containing foods (Images), and the presentation of a real product on consumers’ perceptions of chocolate chip cookies containing cricket protein powder were evaluated sequentially and in combination.

An initial trial of insect-containing foods is a necessary first step toward more regular incorporation into a consumer’s diet. To increase some consumers’ willingness to try edible insects, addressing food neophobia is key [15]. As such, previous experience in consuming insects and processed insect products has been positively associated with future intent [9,16]. Education, exposure to novel food, variety of diet, and social influence have been effective in reducing food neophobia [17,18,19]. For non-neophobic individuals, reluctance to eat insects may be more related to the perceived inappropriateness of consuming insects as food based on cultural conditioning, rather than pure novelty/unfamiliarity [2]. Among those consumers who are willing to try entomophagy, there have been associations with adventurousness and other sensation-seeking traits [3], which encourage new eating experiences [2].

While much of the impetus for expanding entomophagy appeals to the environmental advantages of production compared to traditional livestock [5,6], Western consumers have largely expressed unwillingness to replace meat with insects [4,9]. Rather, snacks and baked goods elicited higher positive trial intent from US consumers [9]. As one of the most familiar food products in the market, chocolate chip cookies can help to establish a positive attitude toward insect-based food products among consumers, which increases their willingness to try [20]. Therefore, chocolate chip cookies (CCCs)—a well-liked and familiar food—were chosen as the vehicle for perceptual data collection in the present research. The objectives of this study were to evaluate the effects of various information conditions (text, images, an actual product, and combinations thereof) related to CCCs containing cricket protein powder on consumers’ perception of the product. We measured panelists’ willingness to consume (WTC), acceptance, and purchase intent (PI) under different information conditions. Conditions were presented sequentially and included increasing levels of information about the insect ingredient’s usage level in the product, physical form, packaging, nutrition, sustainability benefits, and a celebrity endorsement. Perceptions of male and female consumers were also compared, and the predictors of PI were evaluated statistically.

## 2. Materials and Methods

### 2.1. Preparation of Chocolate Chip Cookie (CCC) Samples

The chocolate chip cookies (CCCs) were made following the recipe outlined by Gao et al. [3]. The ingredients for CCCs included Great Value^®^ wheat flour (Bentonville, AR, USA), unsalted butter from Land O’Lakes, Inc. (Arden Hills, MN, USA), chocolate chips from Nestle Toll House^®^ (Solon, OH, USA), sugar from Great Value^®^ (Bentonville, AR, USA), cricket flour (*Acheta domestica*) sourced from Thailand Unique (Udon Thani, Thailand), vanilla extract by McCormick & CO., Inc. (Hunt Valley, MD, USA), Morton salt (Chicago, IL, USA), and whey protein from Grande Custom Ingredients Group (Lomira, WI, USA).

Two different recipes were used for the CCCs, which varied in their inclusion of either cricket flour (WI; comprising 10% cricket powder by the weight of the dough) or whey protein (WO; with 10% whey protein by the weight of the dough). The cookie dough was chilled at 4 °C for two hours and then shaped into 25 g rounds, each 40 mm in diameter, and placed on baking sheets. These were then baked at 180 °C for 12 min, after which they were removed from the oven and allowed to cool to room temperature.

### 2.2. Consumer Sensory Test

The protocol for this study was approved by the Louisiana State University Agricultural Center Institutional Review Board (IRBAG-21-0063). A group of 150 individuals, comprising students, faculty, and staff, was recruited from the Louisiana State University campus in Baton Rouge, LA, USA, for this study. The selection criteria included being 18 years or older, a history of buying and eating chocolate chip cookies, and no allergies to the tested products (including shellfish). The demographic breakdown of the participants was 53% male and 47% female. The age distribution was predominantly 18 to 25 years (77%), with 18% aged 26 to 35, and 5% over 35 years. The racial composition was 46% Caucasian, 15% African American, 19% Asian, 10% Hispanic, and 10% identifying as other races, based on their self-reports.

A digital survey was designed and implemented through the Compusense^®^ five software Release 5.6 by Compusense^®^ Inc., Guelph, ON, Canada. The consumer evaluations were carried out in separate booths at the Sensory Services Lab of Louisiana State University. Participants e-signed the consent form and answered the digital questionnaires, and their responses were captured electronically.

**Figure 1:**
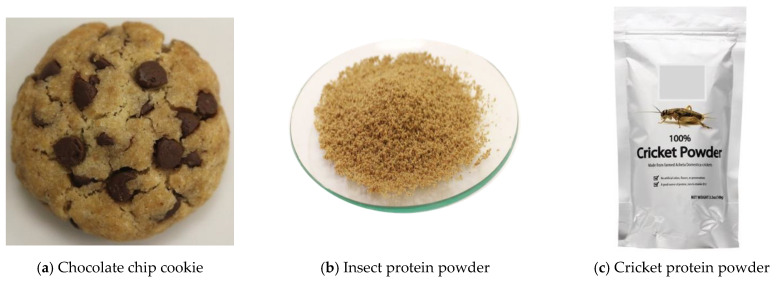

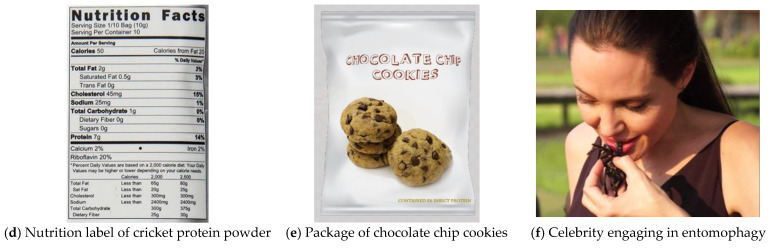
Images presented as components of different information conditions shown in Table 1.

In this study, we tested the effects of different information types and combinations on consumers’ WTC, acceptance, and PI of CCCs. Each condition consisted of either textual information (six variations, Table 1), an image or images (six variations, Figure 1) paired with text (Table 1), an actual cookie presented in a 2 oz cup, or some combination thereof. Each combination of the conditions was considered a treatment. Fifteen treatments were administered sequentially to all consumers, as shown in Table 1, using an online questionnaire. After the presentation of each treatment, consumers rated their WTC (“Would you be willing to consume this product?”/a labeled 5-point scale anchored at “Not at all” and “Extremely”), acceptance (“Based on the above description, please rate your acceptance of the product”/a labeled 9-point hedonic scale anchored at “dislike extremely” and “like extremely”), PI (“Would you like to purchase this product?”/a labeled 5-point scale anchored at “Not at all” and “Extremely”) of the CCC concept presented. The survey was reviewed and filled out by some faculty and students, and they provided comments to improve the quality of the survey prior to collecting actual data.

During the consumer test, cookies made with cricket flour (WI) and without cricket flour (WO) were served to panelists in two-ounce clear plastic cups with transparent lids for visual observation (no tasting involved; Condition 15, Table 1). Photographs of WI and WO were taken with a Canon^®^ Rebel SL1 camera (Melville, NY, USA) separately using the same settings. These photographic images were also used as stimuli in the online consumer test questionnaire (see Figure 1). Images a-e shown in Figure 1 were taken using the same camera and modified using Adobe Photoshop 7.0 software (San Jose, CA, USA), and image f was downloaded from an online news site with permission [21]. The actual cookie samples were served after the panelists finished all 15 information condition questions under text and/or image presentations. No actual taste testing was performed in this study.

### 2.3. Statistical Analyses

We utilized MANOVA to evaluate the impact of the six different informational statements as independent variables, examining their effects on willingness to consume (WTC), acceptance, and purchase intention (PI) as dependent variables. These assessments were carried out separately for both text descriptions and image presentations. Additionally, a one-way ANOVA was conducted, applying Tukey’s studentized range test at a significance level of α = 0.05, to compare the mean scores of WTC, acceptance, and PI. A paired *t*-test was employed to contrast the responses to text descriptions and image presentations associated with the same informational statement. Furthermore, the gender differences across all treatments were investigated using Pearson’s chi-squared test. The regression analysis aimed to pinpoint the factors influencing consumers’ PI, which was measured on a 5-point scale. All statistical analyses were executed using R software, version 3.6.3, and the Statistical Analysis Software^®^ (SAS, 2012 edition).

## 3. Results

### 3.1. WTC, Acceptance, and PI of CCC under Different Information Statements

Consumers’ responses to the text “Chocolate Chip Cookie” (CCC; Condition 1, Table 1), without any mention of the insect or cricket flour, were collected first and used as a baseline for comparison with subsequent information related to insect incorporation. This simple description of a CCC yielded the highest mean scores for WTC (3.73 on a five-point scale), acceptance (7.24 on a nine-point scale), and PI (3.84 on a five-point scale, Table 2) among all other text descriptions. Accordingly, the same text paired with a picture of a chocolate chip cookie (Condition 2, Table 1) produced the highest mean WTC (3.92), acceptance (7.41), and PI (3.66) scores among the text + image conditions. In this case, adding visual information significantly increased WTC but slightly decreased PI.

After being informed that the CCC contained insect protein powder (Condition 3, Table 1), WTC, acceptance, and PI scores demonstrated the steepest drop between any two consecutive conditions, down to 2.11 (a mean drop of 1.81 on the five-point scale), 4.11 (a mean drop of 3.3 on the nine-point scale), and 1.92 (a mean drop of 1.74 on the five-point scale), respectively (Table 2). Presenting an image of the insect protein powder alongside its description (Condition 4, Table 1) further decreased WTC and acceptance scores directionally (but not significantly). Identifying the insect ingredient as cricket (Condition 5, Table 1) further decreased WTC, acceptance, and PI directionally. However, no significant changes in the three affective responses were noticed (Table 2) for Conditions 3 to 8 (Table 1), which informed consumers about the low [5%] usage level of cricket protein and provided pictures of a package of cricket powder.

A significant upward trend was finally observed (Table 2) after the presentation of nutritional information (vitamin B, micronutrients, and all essential amino acids) (Condition 9, Table 1) across all three affective scores, albeit lower than the concept of CCCs without insect (Conditions 1 and 2). Likewise, after presenting text about the sustainability benefits of insect protein (Condition 11, Table 1), WTC and PI increased significantly, with the acceptance score showing a directional increase (Table 2).

In certain cases, pairing an image with text descriptions resulted in significant differences from text alone (Table 2). These differences can be seen between Conditions 1 vs. 2 and Conditions 9 vs. 10, in which the WTC for image presentation was significantly higher than text description. Consumers’ response to information related to a 5% cricket powder incorporation level showed a significant drop in acceptance when the text was paired with images of CCCs side by side with an image of cricket powder (Conditions 7 vs. 8).

Consumers’ responses to the following five conditions paired with the text “Chocolate chip cookie containing 5% insect protein powder” were compared: the text alone (T, Table 3; Condition 7, Table 1); images of a CCC and the principal display panel of a commercially available package of cricket powder (I, Table 3; Condition 8, Table 1); an image depicting an experimenter-designed package displaying “Chocolate Chip Cookies” as the product name, a picture of CCC, and “Containing 5% insect protein” on the bottom of the panel (Pk, Table 3; Condition 13, Table 1); additional text stating “Two billion people eat insects worldwide,” along with an image of the celebrity eating an insect (Cl, Table 3; Condition 14, Table 1); and presentation of a real chocolate chip cookie (for visual evaluation only; R, Table 3; Condition 15, Table 1). According to the comparison of WTC, acceptance, and PI mean scores with the text alone (2.29, 4.14, and 2.10, respectively; T, Table 3), there were significant increases upon the presentation of a CCC package image (up to 2.85, 5.19, and 2.72, respectively; Pk, Table 3), and then the scores dropped again upon the presentation of celebrity endorsement (mean scores of 2.40, 4.33, and 2.19, respectively; Cl, Table 3). However, the highest scores were achieved when consumers were given an actual CCC to observe on each respective affective scale (3.40, 6.17, and 3.07; R, Table 3). After visually examining the actual CCC containing insect protein, all three response scores were rated significantly higher than other informed conditions (Table 3). The actual CCC containing insects received the highest PI at 3.07, while PI scores for the CCC without insect protein were 3.84 (Condition 1, Table 2) and 3.66 (Condition 2, Table 2). Overall, the lowest scores for WTC, acceptance, and PI were found in response to text alone (T), image presentation (I), and the presentation of celebrity endorsement information (Cl).

### 3.2. Differences in WTC, Acceptance, and PI of CCCs by Gender across the Six Information Statements

MANOVA was performed, with female and male consumers separately, to examine the possible association between the text-alone information conditions and the text + image conditions, revealing significant differences between the vectors of WTC, acceptance, and PI (all MANOVA *p* < 0.001) for each gender.

The overall trends observed from the twelve information conditions presented (text and images) were largely preserved when segmenting the current population sample by gender, with a few differences noted between males and females (Table 4). Firstly, females responded more favorably to the concept of a regular (before any mention of insect) CCC both in text (T1) and image presentation (I2). While the mean WTC, acceptance, and PI significantly dropped for both genders upon being given information about insect protein powder within the CCC (T3, I4), the magnitude of the observed changes was greater for females in all three dimensions.

Despite differential initial responsiveness to the concept of CCC, similar trends were observed across genders overall. However, males were more positively influenced by text about nutrition (mean acceptance score of 5.70 vs. 4.87 on the nine-point scale, T9, Table 4) and sustainability benefits (mean acceptance scores of 6.23 vs. 5.44, T11), expressing higher acceptance than females. Scores for males after seeing a nutrition label and sustainability statement (I12) were directionally higher than seeing a nutritional label alone (I10) and were comparable to those of the initial impressions of CCC + its image (I2). For females, however, the series of conditions did not recuperate their mean scores to their original level, before the text or image of insect protein powder was presented (I12 vs. I2, Table 4).

As seen in Table 5, according to Pearson’s chi-squared test, there was no significant difference between males and females based on the five informed conditions (*p* > 0.05). However, females’ responses for WTC, acceptance, and PI after the presentation of an actual CCC (for visual evaluation only; Condition R) were directionally higher than those of males.

### 3.3. Prediction of PI

The regression modeling of consumers’ reported PI was generated using acceptance, WTC, race, informed conditions, and information statements from the entire population sample as well as for each gender separately. In Table 6, the independent variables used to model PI for general, male, and female consumers are presented. In the model for males, the informed conditions were not a significant influencer for the regression, while all the other independent variables were significant regressors.

Table 7 depicts the results of the regression modeling for the PI response. The predictive power (R^2^) indicated that our regression could predict 82% of the total variance of the PI (F = 633.6, df = 16). Acceptance in the regression was significantly associated with the PI (*p* < 0.001), with every unit increase in acceptance scores (on the nine-point scale) associated with a 14% increase in odds of increasing PI (from any category to the next on the five-point scale; e.g., from “slightly” to “moderately”). As shown in Table 7, WTC was the predictor that had the greatest influence on PI; when consumers were more willing to consume the insect-based CCC, they were also more willing to purchase it. The predicted odds of positive PI would nearly double for each unit increase in WTC (OR = 1.92, Table 7). For the race variables, compared to “African American,” “Hispanic/Latino” and “other races” showed a significant effect on PI; as the value of the estimate indicates, Hispanics and “other races” were more willing to purchase CCCs that contained edible insect flour. The image of merchandise packaging (Pk) significantly (positively) affected the PI (OR = 1.14), as did text about the sustainability benefits of insects (Information [11]; OR = 1.19; Table 7).

For males, the predictive power (R^2^) of the regression was 84% (F = 377.7, df = 15). Additionally, the acceptance, WTC, race of “Hispanic” and “other races” significantly influenced the PI. However, in the regression, the informed condition of merchandise packaging (Pk) and the information statement of sustainability compared to the celebrity support of entomophagy did not significantly affect the PI for males. The information statement about adding cricket protein powder to CCC (Information [3]) had a significantly negative influence on the PI, reducing PI odds by 13% (OR = 0.87; Table 7).

The predictive power (R^2^) of the regression was 80% for the model that considered only females’ PI responses (F = 308, df = 15). Similar to the overall population samples, WTC was a predictor with the largest effect on females’ PI; in the regression, a one-unit increase in WTC was associated with a 90% increase in PI odds for females (OR 1.90, Table 7). Compared to African American females, Hispanic females had higher odds of PI for CCCs containing insects. As for information statements, a sustainability benefits claim (Information [12]) would be expected to result in 30% higher odds of PI for women, compared to when they were only informed that the CCC contained cricket flour (Information [3]).

## 4. Discussion and Some Limitations

As the results demonstrate, even a simple mention of an insect ingredient—whether it is actually present or not—can diminish the acceptability of an otherwise well-liked food concept such as brownies [22], or in the present case, CCCs. In such cases, the insect ingredient may be considered a contaminant to the food, which may elicit the emotion of “disgust” [23], even when intentionally incorporated at low levels (e.g., 5% in the CCC, Conditions 7 and 8, Table 1). In the present study, a negative response to entomophagy was evident from the first mention of insects via text (Condition 3, Table 1) and was not alleviated by specifying the insect type as crickets or showing images of the ingredient in its processed form (i.e., cricket powder) or as a packaged product. This result is typical of Western consumers, who have generally shown an aversion to insect-containing foods once the ingredient is revealed [24,25]. Consumer-reported reasons for the avoidance of insect consumption have included general unfamiliarity (often linked to food neophobia) [11,20], poor expectations of sensory quality, and negative emotions such as disgust [3,22,26].

Seemingly more impactful than the information form (text and/or image) was the nature of the information. Significant additive improvements in WTC and PI scores for CCCs were observed when textual information about nutrient composition, and then sustainability, was presented. Other researchers [10,27] found that understanding the health and sustainability benefits of insect-based food could significantly affect consumers’ attitudes and intentions, and it has been suggested elsewhere that separate claims of product health and sustainability benefits are more impactful than integrated messages [28]. In the present study, an image of a nutrition label resulted in a further additive effect on WTC compounded with both text conditions in sequence. Although consumers have found insect flour more favorable to the intact form of the insect [29,30], images depicting cricket powder and a cricket on the package may have provided visual reminders of the insect for consumers in the present study. In general, foods have been deemed more acceptable by consumers when the insect ingredient is unrecognizable or “invisible” in the product or depicted ambiguously or not at all on its packaging [11,12]. However, the label of the production country may also affect consumers’ perception of the insect-based food product. Insect flour labeled from the US received higher expectations than other countries [13]. As such, the present study demonstrated instances of both positive and negative impacts of visual information (i.e., images) on perceptions of an insect-containing product.

In this study, consumers were subjected to sequential informational cues, which elicited similar perceptual trends across all three affective dimensions (WTC, acceptance, and PI). These data indicate that, in the absence of tasting, product information can both negatively (when insects are described and/or presented visually) and positively (when the benefits of insect consumption are communicated) impact consumers’ attitudes toward entomophagy.

Celebrities’ statements about their own dietary choices can exert an effect on consumers’ eating habits [31]. However, in the current study, information about a specific celebrity’s engagement in entomophagy along with her picture did not produce any significant increase across the three affective dimensions measured. Given their social influence, celebrities can help transform unknown products into well-known products; however, in some cases, celebrities with negative public image may also lead to a negative response in consumers’ perception [32,33]. The current results showed that celebrity status alone was not sufficient to change consumers’ perceptions of entomophagy, and it is further hypothesized that different results may have been obtained if a different celebrity was presented.

After seeing a real CCC containing insects, consumers’ WTC, acceptance, and PI were highest among other informed conditions (Table 3 and Table 5). The lack of commercialized insect-based food products in Western countries is one of the many barriers for consumers to practice entomophagy [27] and helps explain consumers’ unfamiliarity. Upon a visual inspection of the CCC, it is possible that negative expectations were alleviated by presenting a familiar product where the insect component was not visible. Providing consumers with opportunities to try insect-based foods could help normalize entomophagy [9]. An appropriate insect-based food product with a tasty flavor that fits the standards of the food product category could improve consumers’ liking and further increase their willingness to buy [34]. In the present case, WTC for CCCs containing cricket powder was highest (among other informed conditions) when presented as a tangible food product rather than more abstract textual or photographic representations.

The most applied factors that could positively affect consumers’ acceptance and adoption of entomophagy include health benefits, global sustainability, familiarity with food products, and gender differences [6,35,36,37]. In the present study, females had higher baseline scores for the CCC but then demonstrated a steeper decline in affective perceptions as information was given about the insect ingredient. For males, providing appropriate nutrition/sustainability benefit information about food containing insects led to similar PI scores as the conventional (insect-free) CCC concept. Other research revealed that women express higher concern about food sustainability [36,38]. In the present study, females’ scores increased directionally in response to the sustainability messaging but were still significantly lower than a CCC without insect protein messaging. This trend is consistent with other gender comparisons related to entomophagy, where males seem more open to adoption overall [2].

Some limitations of this current study included the sample size (*n* = 150) and consumer characteristics. Although the ratio between males and females was about 1:1, the majority of the participants were between 18 and 25 years old (77%), followed by 26–35 (18%), and over 35 (5%) age groups. Even though it has been suggested that a younger generation may be more attracted to insect consumption [6], the current work was not properly designed to test this hypothesis. The expanding age range may cause a decrease in acceptance and purchase intent. Cultural differences were reported to affect consumer liking of CCCs containing cricket flour [39]. Therefore, a future study with a larger sample size and wider demographic range is needed to confirm the findings presented in this study. Regarding the celebrity’s endorsement of insect consumption, only one image of the celebrity was used, so it is most likely possible that different results may have been obtained if a different celebrity or the same celebrity but with different gestures had been presented. In the future, other familiar foods such as breads, crackers, cakes, etc., should be investigated, including other conditions such as actual taste testing, and their results should be compared under the informed conditions used in this study.

## 5. Conclusions

As the study of edible insects gains momentum, issues like unfamiliarity, negative emotional reactions, and food neophobia in Western societies are drawing academic focus. Developing effective strategies for educating about entomophagy and identifying affective factors to mitigate negative perceptions of insect consumption are key steps for going forward. The results from this current study indicated that different presenting formats (text, image(s), and/or combinations as well as presenting an actual product) significantly influenced WTC, acceptance, and PI of CCC. Specifically, showcasing the actual CCC product was effective in lessening the adverse attitudes toward edible insects, whereas displaying images related to insects tended to reinforce negative perceptions. Emphasizing the health and environmental benefits to male consumers could increase their interest in food-containing insects. These insights are crucial for future efforts to promote entomophagy, guiding the selection of information presentation methods that can positively influence consumer choices and enhance the perceived acceptability of insect-based foods. This approach could also attract early adopters to this food innovation. In addition to insect-based food, any innovative food with issues such as negative feelings, distractive appearance, and unpleasant smell could adopt a similar strategy to promote the product.

## Figures and Tables

**Table 1:** Information conditions (text and/or images) presented to consumers related to chocolate chip cookies made with cricket flour.

Condition/ Sequence	Text Presented *	Image(s) ^
1	Chocolate chip cookie.	[none]
2	Chocolate chip cookie.	a
3	Chocolate chip cookie containing insect protein powder.	[none]
4	Chocolate chip cookie containing insect protein powder.	a,b
5	Chocolate chip cookie containing cricket protein powder.	[none]
6	Chocolate chip cookie containing cricket protein powder.	a,c
7	Chocolate chip cookie containing 5% cricket protein powder.	[none]
8	Chocolate chip cookie containing 5% cricket protein powder.	a,c
9	Chocolate chip cookie containing 5% cricket protein powder, which provides vitamin B, micronutrients, and all essential amino acids.	[none]
10	Chocolate chip cookie containing 5% cricket protein powder, which provides vitamin B, micronutrients, and all essential amino acids.	a,c,d
11	Chocolate chip cookie containing 5% cricket protein powder, which provides vitamin B, micronutrients, and all essential amino acids. This product could also support global food sustainability.	[none]
12	Chocolate chip cookie containing 5% cricket protein powder, which provides vitamin B, micronutrients, and all essential amino acids. This product could also support global food sustainability.	a,c,d
13	Chocolate chip cookie containing 5% cricket protein powder in a package.	e
14	Chocolate chip cookie containing 5% cricket protein powder in a package. Two billion people eat insects worldwide. A photo of a celebrity (Angelina Jolie) engaging in entomophagy is presented.	e,f
15 *	Chocolate chip cookie containing 5% cricket protein powder in a package.	e

^ See Figure 1 for actual images. * After the text and image presentation, consumers were presented with an actual chocolate chip cookie (either with or without cricket flour, in a randomized design) for visual evaluation.

**Table 2:** Mean scores for willingness to consume (WTC), acceptance, and purchase intent (PI) of chocolate chip cookies (CCCs) as influenced by different information statements ^1^.

Conditions ^2^	Willingness to Consume		Acceptance		PI	
	Text	Image	Text	Image	Text	Image
1 (Text) and 2 (Image)	3.73 ± 0.99 ^Ad^	3.92 ± 0.86 ^Bc^	7.24 ± 1.56 ^c^	7.41 ± 1.39 ^c^	3.84 ± 1.05 ^Bd^	3.66 ± 0.93 ^Ad^
3 (Text) and 4 (Image)	2.11 ± 1.05 ^a^	2.09 ± 1.03 ^a^	4.11 ± 2.16 ^a^	3.96 ± 2.22 ^a^	1.92 ± 1.01 ^a^	1.97 ± 1.01 ^a^
5 (Text) and 6 (Image)	1.97 ± 1.06 ^a^	2.02 ± 1.05 ^a^	3.61 ± 2.23 ^a^	3.55 ± 2.23 ^a^	1.85 ± 1.05 ^a^	1.91 ± 1.03 ^a^
7 (Text) and 8 (Image)	2.29 ± 1.06 ^a^	2.25 ± 1.09 ^a^	4.14 ± 2.22 ^Ba^	3.91 ± 2.25 ^Aa^	2.1 ± 1.03 ^a^	2.09 ± 1.08 ^a^
9 (Text) and 10 (Image)	2.95 ± 0.92 ^Ab^	3.04 ± 0.99 ^Bb^	5.31 ± 2.01 ^b^	5.45 ± 2.01 ^b^	2.71 ± 1.03 ^b^	2.79 ± 1.07 ^b^
11 (Text) and 12 (Image)	3.29 ± 1.05 ^c^	3.32 ± 1.07 ^b^	5.86 ± 2.00 ^b^	5.98 ± 1.94 ^b^	3.18 ± 1.09 ^c^	3.25 ± 1.08 ^c^

^1^ WTC: willingness to consume, a 5-point scale; acceptance: a 9-point scale; PI: purchase intent, a 5-point scale. ^2^ Odd-numbered conditions contained text alone (Text); even-numbered conditions contained images (Image). See Table 1 and Figure 1 for descriptions of each condition. ^a–d^ The different letters following means and standard deviation values in the same column are significantly different based on the ANOVA test (*p* < 0.05). ^A,B^ The different superscripts following the means and standard deviation values of WTC, acceptance, or PI stand for significant differences between the text description alone (Text) and the image presentation (Image) under the paired *t*-test (*p* < 0.05).

**Table 3:** Mean scores ^1^ for willingness to consume (WTC), acceptance, and purchase intent (PI) of chocolate chip cookies (CCCs) as influenced by different informed conditions.

Informed Condition ^2^	WTC	Acceptance	PI
T	2.29 ± 1.06 ^a^	4.14 ± 2.22 ^a^	2.10 ± 1.03 ^a^
I	2.25 ± 1.09 ^a^	3.91 ± 2.25 ^a^	2.09 ± 1.08 ^a^
Pk	2.85 ± 1.10 ^b^	5.19 ± 2.21 ^b^	2.72 ± 1.19 ^b^
Cl	2.40 ± 1.14 ^a^	4.33 ± 2.21 ^a^	2.19 ± 1.12 ^a^
R	3.40 ± 1.04 ^c^	6.17 ± 1.89 ^c^	3.07 ± 1.09 ^c^

^1^ The different letters following means and standard deviation values in the same column are significantly different based on the ANOVA test (*p* < 0.05). ^2^ T = the text “Chocolate chip cookie containing 5% cricket protein powder” alone; I = images of a CCC and of the principal display panel of a commercially available package of cricket powder; Pk = an image depicting an experimenter-designed package displaying “Chocolate Chip Cookies” as the product name, a picture of CCC, and “Containing 5% insect protein” on the bottom of the panel; Cl = Pk + an additional text stating “Two billion people eat insects worldwide,” along with an image of the celebrity eating an insect; R = presentation of an actual chocolate chip cookie (for visual evaluation only). See Table 1 and Figure 1 for descriptions of each Condition.

**Table 4:** Mean scores for willingness to consume (WTC), acceptance, and purchase intent (PI) of chocolate chip cookies (CCCs) for males and females as influenced by different information statements.

Condition ^1^	WTC		Acceptance		PI	
	Male	Female	Male	Female	Male	Female
T1	3.54 ± 0.99 ^aA^	3.96 ± 0.96 ^aB^	6.99 ± 1.62 ^aA^	7.53 ± 1.45 ^aB^	3.23 ± 0.95 ^abA^	3.77 ± 1.09 ^aB^
T3	2.26 ± 1.03 ^c^	1.94 ± 1.05 ^c^	4.29 ± 2.15 ^c^	3.90 ± 2.15 ^cd^	2.06 ± 0.99 ^c^	1.76 ± 1.03 ^c^
T5	2.21 ± 1.01 ^c^	1.96 ± 1.03 ^c^	4.20 ± 2.14 ^c^	3.69 ± 2.29 ^d^	2.08 ± 0.98 ^c^	1.86 ± 1.04 ^c^
T7	2.41 ± 1.05 ^c^	2.16 ± 1.06 ^c^	4.42 ± 2.21 ^c^	3.81 ± 2.22 ^d^	2.16 ± 1.02 ^c^	2.03 ± 1.04 ^c^
T9	3.01 ± 0.91 ^b^	2.87 ± 0.93 ^b^	5.70 ± 1.88 ^bB^	4.87 ± 2.09 ^bcA^	2.81 ± 1.01 ^b^	2.60 ± 1.04 ^b^
T11	3.34 ± 1.03 ^ab^	3.23 ± 1.07 ^b^	6.23 ± 1.98 ^abB^	5.44 ± 1.96 ^bA^	3.29 ± 1.08 ^a^	3.06 ± 1.10 ^b^
I2	3.79 ± 0.91 ^aA^	4.07 ± 0.79 ^aB^	7.20 ± 1.54 ^aA^	7.66 ± 1.17 ^aB^	3.46 ± 0.94 ^aA^	3.89 ± 0.88 ^aB^
I4	2.21 ± 1.01 ^c^	1.96 ± 1.03 ^c^	4.20 ± 2.14 ^c^	3.69 ± 2.29 ^c^	2.08 ± 0.98 ^c^	1.86 ± 1.04 ^c^
I6	2.06 ± 1.04 ^c^	1.97 ± 1.06 ^c^	3.72 ± 2.18 ^c^	3.36 ± 2.28 ^c^	2.02 ± 1.01 ^c^	1.77 ± 1.04 ^c^
I8	2.38 ± 1.07 ^c^	2.11 ± 1.11 ^c^	4.09 ± 2.22 ^c^	3.71 ± 2.28 ^c^	2.22 ± 1.06 ^c^	1.93 ± 1.09 ^c^
I10	3.10 ± 0.99 ^b^	2.97 ± 0.99 ^b^	5.74 ± 1.95 ^b^	5.13 ± 2.04 ^b^	2.89 ± 1.10 ^b^	2.67 ± 1.02 ^b^
I12	3.39 ± 1.11 ^ab^	3.24 ± 1.03 ^b^	6.35 ± 1.94 ^abB^	5.56 ± 1.86 ^bA^	3.34 ± 1.12 ^ab^	3.16 ± 1.03 ^b^

^1^ Odd-numbered conditions contained text alone (Text, T); even-numbered conditions contained images (Image, I). See Table 1 and Figure 1 for the description of each condition. ^a–d^ Within T or I condition, different letters following means and standard deviation values in the same column are significantly different (*p* < 0.05). ^A,B^ The different superscripts following the means and standard deviation values of WTC, acceptance, or PI, stand for significant differences between males and females according to Pearson’s chi-squared test (*p* < 0.05).

**Table 5:** Mean scores for willingness to consume (WTC), acceptance, and purchase intent (PI) of chocolate chip cookies (CCCs) by gender as influenced by different informed conditions.

Informed Condition ^1^	WTC		Acceptance		PI	
	Male	Female	Male	Female	Male	Female
T	2.79 ± 1.13 ^b^	2.69 ± 1.25 ^b^	5.30 ± 2.27 ^ab^	4.87 ± 2.44 ^b^	2.60 ± 1.13 ^ab^	2.51 ± 1.27 ^bc^
I	2.82 ± 1.20 ^b^	2.72 ± 1.27 ^b^	5.22 ± 2.38 ^b^	4.85 ± 2.51 ^b^	2.67 ± 1.19 ^ab^	2.55 ± 1.28 ^b^
Pk	2.54 ± 1.11 ^b^	2.24 ± 1.15 ^c^	4.59 ± 2.17 ^b^	4.03 ± 2.23 ^b^	2.29 ± 1.08 ^b^	2.07 ± 1.16 ^c^
Cl	2.86 ± 1.06 ^ab^	2.83 ± 1.15 ^b^	5.38 ± 2.15 ^ab^	4.99 ± 2.26 ^b^	2.75 ± 1.13 ^ab^	2.69 ± 1.27 ^ab^
R	3.35 ± 1.07 ^a^	3.45 ± 1.00 ^a^	6.05 ± 1.90 ^a^	6.30 ± 1.88 ^a^	2.95 ± 1.10 ^a^	3.21 ± 1.07 ^a^

^1^ Refer to Table 3 footnote for the description of informed conditions. ^a–c^ The different letters following means and standard deviation values in the same column are significantly different (*p* < 0.05).

**Table 6:** Independent variables for purchase intent (PI) prediction of chocolate chip cookies.

Independent Variables	General		Male		Female	
	F Value	Pr(>F)	F Value	Pr(>F)	F Value	Pr(>F)
Acceptance	8875.9	<0.001	5059.8	<0.001	3954.97	<0.001
WTC	1187.0	<0.001	578.26	<0.001	601.46	<0.001
Race	5.88	<0.001	2.63	0.03	5.91	<0.001
Informed condition	2.55	0.04	0.34	0.85	2.89	0.02
Information statements	7.37	<0.001	3.22	0.01	5.63	<0.001

**Table 7:** Odds ratio estimates to predict the purchase intent of chocolate chip cookies.

Response = Purchase Intent for General Population Sample		
Predictors ^^^	Odds Ratio (OR)	*p*-Value
(Intercept)	-	0.803
**Acceptance**	**1.14**	**<0.001**
**WTC**	**1.92**	**<0.001**
Race [Asian]	1.05	0.225
Race [Caucasian]	1.03	0.31
**Race [Hispanic]**	**1.20**	**<0.001**
**Race [Other]**	**1.15**	**0.002**
**Informed condition [Pk]**	**1.14**	**0.033**
Informed condition [I]	1.05	0.365
Informed condition [R]	0.99	0.883
Informed condition [T]	1.01	0.852
Information [3]	0.94	0.169
Information [5]	1.00	0.946
Information [7]	0.97	0.56
Information [9]	0.98	0.659
**Information [11]**	**1.19**	**<0.001**
R^2^/ R^2^ adjusted	0.819/0.818	
Response = Purchase Intent for Males		
Predictors ^^^	Odds Ratio (OR)	*p*-value
(Intercept)	-	0.66
**Acceptance**	**1.15**	**<0.001**
**WTC**	**1.93**	**<0.001**
Race [Asian]	1.04	0.438
Race [Caucasian]	1.08	0.056
**Race [Hispanic/Latino]**	**1.13**	**0.029**
**Race [Other]**	**1.21**	**0.003**
Informed condition [Pk]	1.11	0.26
Informed condition [I]	1.02	0.784
Informed condition [R]	1.03	0.733
Informed condition [T]	1.01	0.92
**Information [3]**	**0.87**	**0.045**
Information [5]	0.90	0.138
Information [7]	0.92	0.213
Information [9]	0.89	0.07
Information [11]	1.08	0.2
R^2^/R^2^ adjusted	0.846/0.843	
Response = Purchase Intent for Females		
Predictors ^^^	Odds Ratio (OR)	*p*-value
(Intercept)	-	0.952
**Acceptance**	**1.14**	**<0.001**
**WTC**	**1.90**	**<0.001**
Race [Asian]	1.06	0.302
Race [Caucasian]	1.00	0.936
**Race [Hispanic]**	**1.32**	**<0.001**
Race [Other]	1.12	0.089
Informed condition [Pk]	1.16	0.064
Informed condition [I]	1.07	0.342
Informed condition [R]	0.96	0.603
Informed condition [T]	1.01	0.886
Information [3]	1.00	0.995
Information [5]	1.08	0.206
Information [7]	1.02	0.715
Information [9]	1.07	0.247
**Information [11]**	**1.30**	**<0.001**
R^2^/R^2^ adjusted	0.796/0.793	

^^^ Predictors (regressors) in bold typeface were significant to the regression model (α = 0.05). Refer to Table 3 footnote for the description of informed condition Pk, I, R, T. Refer to Table 1 for the description of information statements (Conditions 3, 5, 7, 9, and 11).

## Data Availability

Data are contained within the article.
